# Human Anthrax in Dolj County, Romania—A Series of Three Cases

**DOI:** 10.3390/pathogens10060644

**Published:** 2021-05-23

**Authors:** Florentina Dumitrescu, Eugen Florin Georgescu, Lucian Giubelan, Vlad Pădureanu, Andreea Cristina Stoian, Viorica Dincă, Milena Georgescu, Livia Dragonu, Daniela Marinescu

**Affiliations:** 1 Department of Infectious Disease, Faculty of Medicine, University of Medicine and Pharmacy of Craiova, 200349 Craiova, Romania; dumitrescu_florentina@yahoo.com (F.D.); ligiubelan@yahoo.com (L.G.); andreea_plr@yahoo.com (A.C.S.); livia_dragonu@yahoo.com (L.D.); 2 Department of General Surgery, Faculty of Medicine, University of Medicine and Pharmacy of Craiova, 200349 Craiova, Romania; dtmarinescu@yahoo.com; 3 Department of Internal Medicine, Faculty of Medicine, University of Medicine and Pharmacy of Craiova, 200349 Craiova, Romania; 4 Department of Intensive Care, Victor Babes Clinical Hospital of Infectious Diseases and Pneumology Craiova, 200515 Craiova, Romania; dr.viodinca@yahoo.com; 5 Department of Plastic Surgery, Emergency Clinical County Hospital, 200642 Craiova, Romania; dr.milenageorgescu@gmail.com

**Keywords:** cutaneous anthrax, compartment syndrome, disease, animals

## Abstract

*Bacillus anthracis* is the causative agent of anthrax, primarily a disease of herbivorous animals, which can be accidentally transmitted to humans. Three cases of cutaneous human anthrax were recorded in August 2020 in Dolj county, Romania. These cases included livestock farmers (husband and wife, as well as a man from their entourage). The women presented malignant edema, which required surgery for compartment syndrome; and the men presented the common form of cutaneous anthrax. According to the laboratory investigation, two cases complied with the criteria in the case definition. All cases were successfully treated with antibiotics and the women received reconstructive plastic surgery of the skin defects, restoring normal hand function. The contact with sick animals was ruled out by the health authorities concluding that it was the contamination of pre-existing skin lesions with *B. anthracis* spores from the soil, the anthracogenic area.

## 1. Introduction

Anthrax is an acute bacterial zoonotic disease that affects mammals (especially herbivores) and caused by a Gram-positive bacillus that forms environmentally resistant spores, *Bacillus anthracis*. Anthrax continues to be a problem for developing countries, being endemic in some parts of the world, such as Central Africa or African countries [1], but it occurs rarely in Europe. The disease is transmitted to humans through contact with infected animals, exposure to contaminated animal products, contact with spore contaminated soil, or inhalation of spores or possibly through bites of insects that have fed on sick animals [1]. The tissue lesions produced by *B. anthracis* are caused by its three major virulence factors (lethal toxin, edematous toxin and capsule) that are encoded by plasmids (pOX1 and pOX2) and activated only in the vegetative forms of the bacterium [2]. The possibility of horizontal transmission of these plasmids between bacterial strains was observed by their isolation in *Bacillus cereus* strains (*B. cereus* biovar *anthracis*), which caused severe cases of anthrax-like disease in humans and primates [3].

Depending on the route of exposure, *B. anthracis* infection in humans can be most commonly acquired by the penetration of spores through the skin (cutaneous anthrax) or rarely through the mucous membrane (gastrointestinal anthrax) or by inhalation of spores into the lungs (pulmonary anthrax) [4]. Cutaneous anthrax (malignant pustule and malignant edema) accounts for more than 95% of human cases of natural infection [1,4]. In the case of malignant edema, the risk of complications is increased by the development of bacteremia, local superinfection or the very rare possibility of acute compartment syndrome [5]. The prognosis of the disease depends on the clinical form, mortality being high in visceral anthrax. Inhalation anthrax and secondary systemic infection (e.g., sepsis, haemorrhagic leptomeningitis) have a mortality rate approaching 100%. Cutaneous anthrax may have a fatality rate of up to 20% if left untreated, but lethality is reduced to <1% by therapy [1].

All clinical forms of anthrax require the administration of antibiotic treatment, the combination of monoclonal antibodies being recommended in inhaled anthrax [4,6]. From a surgical point of view, in the uncomplicated cutaneous anthrax, a conservative attitude is recommended because the surgical intervention in the acute period can determine a possible bacteremia [4,6]. However, treatment decisions must be made on the basis of the individual presentation of each case [5]. In this sense, the paper aims to present some particularities of diagnosis and treatment for cutaneous anthrax, by presenting case reports that discuss the possibility of this disease in atypical form.

## 2. Results

### 2.1. Case 1

**Figure 1:**
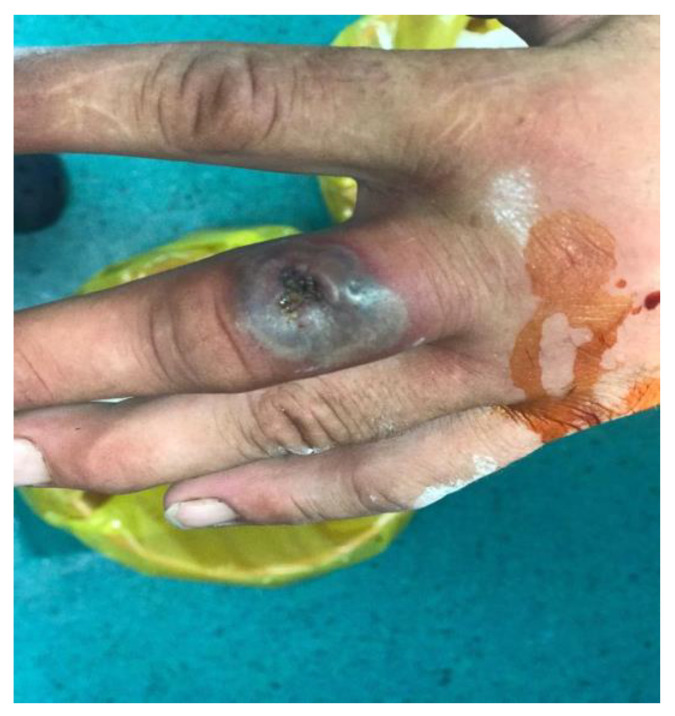
Case 1 at admission. Skin lesions of anthrax on hand. Cutaneous anthrax showing the typical black eschar with surrounding edema.

A 44-year-old male goat-breeding farmer presented himself to the hospital on 7 August 2020 for a 10-day insect bite wound (affirmative) at the level of the third finger of the left hand and for left forearm lymphangitis. Prior to the presentation, Tetracycline was administered 4 times per day for 3 days. The clinical aspect of the lesion suggested a malignant pustule (Figure 1) and raised suspicion of anthrax. 

The patient was hospitalized and was given intravenous Penicillin G and oral Ciprofloxacin, with a favorable evolution. The culture of plague secretion revealed the presence of *Staphylococcus aureus*. Serology for *B. anthracis* showed seroconversion (negative at admission and positive after 12 days). The patient’s wife was admitted to hospital on the same day, a few hours later, for right upper limb edema.

### 2.2. Case 2

A 44 years old female farmer, the latter patient’s wife, was hospitalized on 7 August 2020 for swelling of the right upper limb, rapidly expanding in a few hours, causing compartment syndrome. The swelling was preceded by an insect bite (affirmative) at the volar surface of the right forearm. 

On admission, the patient presented herself with modified general status and fever; moderate edema of the right hand; massive edema of the right forearm and arm with blisters at this level. Within a few hours after admission, she developed compartment syndrome, absent a pulse in the ulnar artery and weak pulse in the radial artery, as well as pale and cold fingers of the right hand. The cardio-respiratory state was stable. Antibiotic treatment was associated with urgent fasciotomy. 

**Figure 2:**
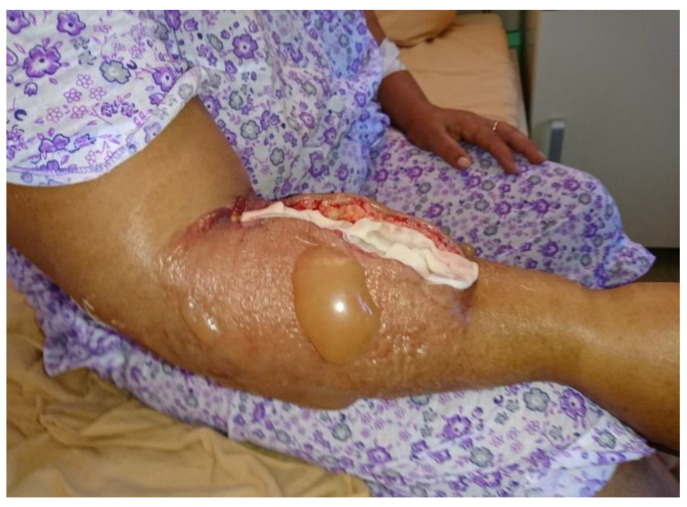
Case 2 after fasciotomy. Significant edema of the right forearm and arm, right forearm blisters.

The immediate postoperative evolution (Figure 2) was favorable, with pulse present in the ulnar and radial artery, capillary refill of the fingers, and mild remission of the edema and normal cardiorespiratory assessment.

She was transferred to our infectious diseases department and continued with intravenous Meropenem 1 g × 3/day, Vancomycin 1 g × 2/day, po Ciprofloxacin 750 mg × 2/day, corticosteroid, vasodilators (Pentoxifilin), analgesics, anticoagulants (low molecular weight heparin), daily local dressings with slow favorable evolution.

**Figure 3:**
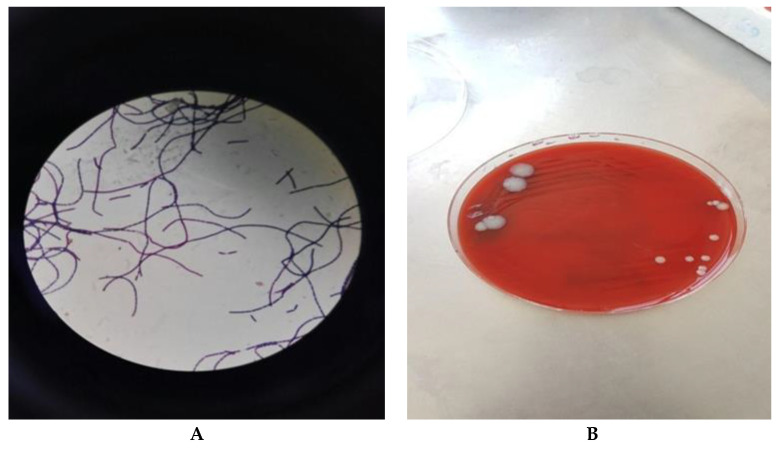
Polychrome methylene blue stain of *B. anthracis* (**A**) and culture on blood agar (**B**)—case 2.

The local laboratory showed *B. anthracis* on Gram stained smear and in blood agar culture performed from secretions harvested from the surgical wound (Figure 3). The results were confirmed by the “Cantacuzino” National Reference Laboratory for Zoonotic Infections, Bucharest. The antibiogram for *B. anthracis* revealed sensitivity to Penicillin, Ciprofloxacin, Tetracycline. Blood culture were negative. PCR for *B. anthracis* was not available.

Serology for *B. anthracis* was negative at admission and positive after 12 days. 

Biological: Leukocytosis with neutrophilia, inflammatory markers with high values (Table 1).

**Figure 4:**
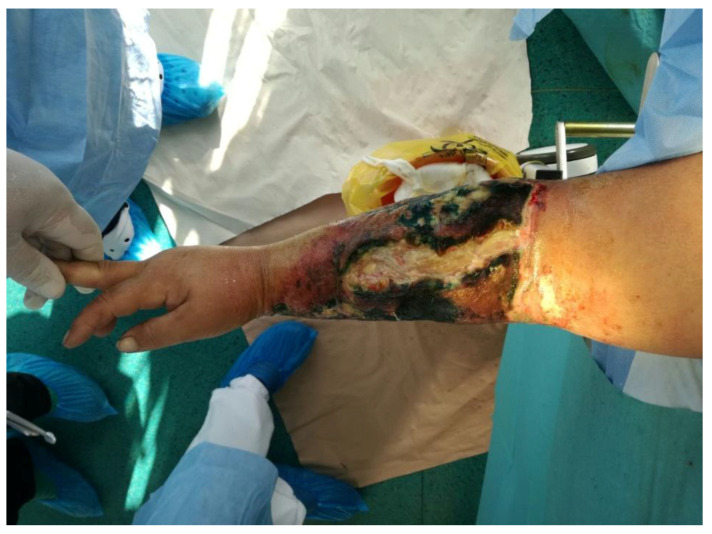
Case 2 following tangential escharotomy.

After 17 days post-admission, a tangential escharotomy was performed (Figure 4), followed by reconstructive plastic surgery of skin defects and restoration of normal hand functions.

### 2.3. Case 3

**Figure 5:**
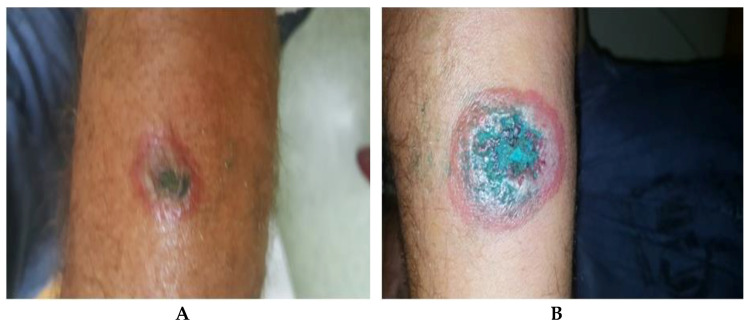
Case 3 at admission. (**A**) Black crust, surrounded by edema and blisters, at the level of the left forearm. (**B**) Similar lesion to the middle 1/3 in front of the right forearm, smaller in size.

On 14 August, 2020, another 41-year-old patient was admitted to the hospital for a malignant pustule suspicion (Figure 5), showing two skin lesions on both forearms, about one week old, after cleaning in an unused barn and skinning a goat from the farmers presented earlier.

The first cutaneous lesion appeared in the medium third, volar surface of the left forearm, approximately 5 days after the animal slaughter, and in the next 2–3 days at the counter lateral forearm. Prior to admission, the patient had self-administered Tetracycline 4 times per day for 3 days, and applied locally methylene blue, and hyaluronic acid and chlorhexidine gluconate. The smear and culture from the lesions revealed *S. aureus* methicillin-resistant, and serology for *B. anthracis* was negative at admission and after 12 days. The patient followed treatment with oral Ciprofloxacin 750 mg × 2/day, intravenous Penicillin G 2 MUI × 4/day and Vancomycin 1 g × 2/day with favorable evolution.

## 3. Discussion

Anthrax is a zoonotic disease endemic in some parts of the world, such as Central Africa or African countries [1,7], but it occurs rarely in Europe. According to ECDC data, human anthrax is sporadic in Romania, being associated mainly with occupational exposure (last it was reported in 2014: Two cases of cutaneous anthrax with epidemiological link, without laboratory confirmation [8]. 

In Romania, the clinical suspicion of anthrax is immediately announced by telephone to the human and veterinary medical authorities, in order to quickly carry out epidemiological and etiological investigations. The case definition is one recommended by the EU [1]. The diagnosis of anthrax being based on clinical criteria, and an epidemiological link for the probable case, respectively clinical and laboratory criteria (culture or detection by PCR of *B. anthracis* in a clinical specimen) for the confirmed case. The case definition adopted in the EU does not include laboratory criteria and serology for *B. anthracis* as the CDC does [9].

The source of the animal infection was not identified for any of the three cases. After examining livestock in the area, representatives of the Sanitary Veterinary Directorate in the area, have not found any diseased animals, considering the contamination of pre-existing skin lesions with *B. anthracis* spores from the soil [10], the area being anthracogenic.

For our patients, we corroborated the epidemiological, clinical and biological data (Table 2) for positive diagnosis.

One case showed severe cutaneous anthrax with the development of acute compartment syndrome that required emergency surgery. The surgical treatment is not recommended in the acute phase, due to the risk of bacteraemia [1,11], but the presence of compartment syndrome requires urgent fasciotomy, in order to save the affected limb. Extensive arm edema, which is considered a risk for compartment syndrome, has rarely encountered [12,13]. So far, there have only been a few reported cases in the literature where surgical treatment, combined with medical treatment, have contributed to the decrease in mortality [11,14]. Emergency fasciotomy rescued the patient’s upper limb without disseminating the infection. The isolated *B. anthracis* showed sensitivity to antibiotics used as preferred therapy (Penicillin, Ciprofloxacin, Tetracycline), Penicillin resistant cases being a rarity [1,15].

Previous antibiotic treatment may result in a negative result of the smear and culture [2,16]. The lack of culture isolation of *B. anthracis* from cutaneous lesions has been reported by some authors [1,17] after 24–48 h since initiation of antibiotic treatment, in these cases being difficult to confirm the diagnosis in the absence of accessible tests in specialized laboratories (serological, immunohistochemistry, tests for specific protective antigen and chain polymerase) [1].

Dynamic serological tests were positive in one of the patients in the absence of positive cultures from the cutaneous lesions (Table 3). There are authors who consider that a negative serological test does not rule out the diagnosis of anthrax if there is a characteristic clinical picture in a suggestive epidemiological context [8,18]. The absence of seroconversion was observed due to early treatment during infection, even in bacteriologically confirmed cases [8,12,19,20,21].

## 4. Materials and Methods

In August 2020, three cases with a suspicion of cutaneous anthrax were recorded in the rural area of Dolj County, Romania. Patients were at an occupational risk, originating from an area where seven years earlier, some anthrax cases were recorded. Two of the cases have been confirmed and reported by the health authorities in Dolj County to the National Center for Communicable Diseases Surveillance and Control. The third case with clinical suspicion of cutaneous anthrax did not comply with the EU criteria [1] of the case definition. 

We analyzed all epidemiological, clinical and biological data recorded in patients’ medical records. 

## 5. Conclusions

Although anthrax is rarely encountered in current medical activity, the recognition of lesions was important for the rapid initiation of investigations, applying epidemiological measures and appropriate treatment. High performance laboratory techniques can supplement investigations in negative culture cases. 

## Figures and Tables

**Table 1:** Biological parameters case 2.

Laboratory Test Report	Normal Range	Unit	At Admission	After 4 Days	After 7 Days	After 10 Days	At Discharge
White blood cells (WBC)	4.0–9.0	×10^3^/mm^3^	13.4	22.3	20.5	15.8	10.9
Mature neutrophils	34–67	%	77	79	76	55	65
Immature neutrophils	0–3.0	%	7	8	12	12	4
Eosinophils	0–5	%	0	0	1	3	7
Basophils	0–1	%	0	0	0	0	1
Lymphocytes	20–50	%	12	8	7	24	16
Monocytes	4–8	%	4	5	4	6	7
Red blood cells (RBC)	3.50–4.50	×10^6^/mm^3^	4.91	4.52	4.04	3.97	3.84
Hemoglobin (HGB)	11.5–14.5	g/dL	14.5	13.4	12.1	12	11.4
Hematocrit (HCT)	35.0–43.0	%	46.4	42.9	38.5	34.6	34.6
Mean corpuscular volume (MCV)	75.0–95.0	μm^3^	95	95	95	87	90
Mean corpuscular hemoglobin (MCH)	26.0–32.0	pg	29.5	29.6	30	30.3	29.7
Mean corpuscular hemoglobin concentration (MCHC)	31.0–37.9	g/dL	31.2	31.2	31.5	34.8	32.9
Red cell distribution width (RDW)	11.0–16.0	%	11.3	11.4	11.4	11.3	12.1
Platelets (PLT)	150–450	×10^3^/mm^3^	187	167	213	291	276
Mean platelet volume (MPV)	9.0–13.0	μm^3^	8.1	7.7	7.7	7.3	7.6
Erythrocyte sedimentation rate (ESR)	1–12/1 h	mm/hour	16	80			20
	4–25/2 h		33	100			44
Fibrinogen	150–400	mg/dL		451	251		
INR			0.98	1.01	0.97	0.99	0.96
GPT	10–35	U/L	20.3	24.6	72.3	79.3	78.9
GOT	0–32	U/L	23.6	24	55	34.6	36.2
Na^+^	136–145	mmol/L	130.1	127.1	129.8	127.6	129.5
K^+^	3.30–5.10	mmol/L	3.53	3.79	3.89	4.07	4.73
Serum total protein	66–87	g/L		47.7	46.2		68
Blood glucose level	70–115	mg/dL	126.2	127	121.6	64.8	89.3
Serum creatinine	0.50–0.90	mg/dL	0.68	0.75	0.63	0.50	0.48
Serum urea	10–50	mg/dL	30.3	27	38.8	51.9	36.8
Total bilirubin	0–1	mg/dL	0.25	0.2	0.14	0.17	
C-Reactive Protein	<10	mg/L		12	<6		
Procalcitonin	<0.25	ng/mL		<0.25			

**Table 2:** Positive diagnosis for all patients.

Case	Clinical Aspect	Gram Smear/Culture	Serological Tests in Dynamics	Epidemiological Link
1	Cutaneous anthrax	Negative	Positive	Yes
2	Malignant edema	Positive	Positive	Yes
3	Cutaneous anthrax	Negative	Negative	Yes

**Table 3:** Bacteriological and serological tests for all cases.

Case	Culture	Gram Smear	Previous Antibiotic	Antibodies anti *B. anthracis* at Admission	Antibodies anti *B. anthracis* after 10–14 (Days)
1	Negative (a)	Negative (a)	Yes	Negative (b)	Positive (T = 1/10) (b)
2	Positive (a, b)	Positive (a, b)	No	Negative (b)	Positive (T = 1/10) (b)
3	Negative (a)	Negative (a)	Yes	Negative(b)	Negative (b)

a—performed at the local laboratory. b—performed at the “Cantacuzino” National Reference Laboratory for Zoonotic Infections, Bucharest, Romania.

## Data Availability

Not applicable.
